# Mental health and health-related behaviors in Chinese college students: the role of physical education

**DOI:** 10.3389/fpubh.2025.1688244

**Published:** 2025-10-16

**Authors:** Qing Xu, Zan Zhou, Zhongfei Tian

**Affiliations:** ^1^Business School, Shaoxing University, Shaoxing, Zhejiang, China; ^2^Institute of Physical Education, Shaoxing University, Shaoxing, Zhejiang, China; ^3^Beijing Health Vocational College, Beijing, China

**Keywords:** mental health, physical education, students, physical activity, obesity, sleep

## Abstract

**Background:**

University physical education (PE) class may promote healthier behaviors and better mental health, yet evidence on their impact in higher education is limited. This study examined associations between PE participation, mental health, health-related behaviors, and sleep quality among Chinese university students.

**Methods:**

A cross-sectional survey was conducted among 2,117 s-year undergraduate students, using proportional stratified sampling in 2024. Validated instruments were used to collect data: demographics and anthropometrics (self-report height and weight to calculate BMI); International Physical Activity Questionnaire–Short Form (IPAQ-SF) for total weekly physical activity; General Health Questionnaire–12 (GHQ-12) for mental health; Pittsburgh Sleep Quality Index (PSQI) for sleep quality; and self-reported screen time. To objectively assess activity levels during PE lessons, ActiGraph wGT3X-BT accelerometers were used. Logistic and multiple linear regression analyses identified associations and predictors of mental health, health-related behaviors, and MVPA proportion during PE class.

**Results:**

In linear models, total physical activity (*β* = 0.17, *p* = 0.003), greater MVPA during PE (*β* = 0.19, *p* = 0.003), and shorter screen time (*β* = −0.14, *p* = 0.009) predicted better mental health. On average, students spent 48.6% of PE lesson time in MVPA. A higher MVPA proportion was associated with lower odds of obesity (OR = 0.41, *p* < 0.001), physical inactivity (OR = 0.38, *p* < 0.001), and poor sleep quality (OR = 0.72, *p* = 0.004). Male students had greater odds of obesity (OR = 3.14, *p* < 0.001) and smoking (OR = 2.02, *p* = 0.001), while females reported poorer sleep. Predictors of MVPA proportion in PE included higher BMI (*β* = −0.11, *p* < 0.001), female gender (*β* = −0.14, *p* < 0.001), poorer sleep quality (*β* = −0.09, *p* = 0.003), and lower mental health (*β* = 0.11, *p* = 0.002).

**Conclusion:**

PE class may provide an important context for promoting health-related behaviors and mental health among university students. However, given the cross-sectional design, causal relationships cannot be inferred. Longitudinal and intervention studies are needed to confirm these findings and clarify underlying mechanisms.

## 1 Introduction

Mental health during young adulthood is a critical public health concern, influencing long-term well-being, academic performance, and social functioning. University students face unique stressors, including heavy academic workloads, social pressures, and the transition to independent living, which can increase their vulnerability to anxiety, depression, and sleep disorders (1). Recent studies suggest that these problems are highly prevalent among Chinese students. For example, a 2024 national survey found that 16.3% of undergraduates had positive psychological symptoms, and the prevalence of these symptoms was higher among girls (17.5%) than boys (14.7%) (2). Sleep quality is also of concern: in a large sample of 6,363 university students in China, more than 60% reported having poor sleep, and poor sleep was significantly associated with an increased risk of psychological symptoms (3). In addition to mental health and sleep, lifestyle behaviors such as screen time, physical activity, and weight status are increasingly recognized as important determinants of student health. Excessive screen time is common in this population, with students spending an average of more than 5 h per day using digital devices (4). Long screen time combined with inadequate physical activity has been associated with a significantly increased risk of psychological symptoms compared with students who are more physically active and use screens less (5). Increasing rates of overweight and obesity among Chinese youth and adolescents also exacerbate these concerns, indicating a shift toward a sedentary lifestyle and unhealthy behaviors (5). The combination of these factors highlights the urgent need for interventions that simultaneously target the physical and mental health of university students.

Physical activity is a well-established contributor to psychological well-being, with evidence suggesting that regular engagement can enhance mood, reduce stress, alleviate anxiety and depressive symptoms, and improve cognitive functioning (6, 7). Within the university context, structured physical education (PE) classes provide an organized setting that may encourage such activity. Although PE primarily targets physical fitness, it can also create opportunities for students to engage in moderate-to-vigorous physical activity (MVPA), experience social interaction, and develop self-efficacy, all of which may indirectly support mental health (8, 9).

Beyond its potential effects on psychological outcomes, PE participation may influence healthy lifestyle behaviors such as sleep patterns, sedentary screen time, and body weight management (10). These behaviors can act as mediators or moderators in the relationship between physical activity and mental health, highlighting the multifaceted role of PE in promoting holistic well-being.

Guided by Social Cognitive Theory (11), this study considers how engagement in PE classes might strengthen students’ confidence to maintain an active lifestyle, facilitate observational learning from peers, and provide positive reinforcement through physical and mental health benefits. These psychosocial mechanisms may extend beyond the classroom, influencing sleep, sedentary behavior, and other lifestyle factors that collectively contribute to mental health outcomes.

### 1.1 Objectives

The present study was designed with three main objectives:

1. To examine the association between participation in PE classes—measured by the proportion of time spent in MVPA—and mental health among Chinese university students.
2. To assess the associations between participation in PE classes and a range of health-related behaviors, including sleep quality, screen time, BMI, obesity, and smoking.
3. To identify predictors of the proportion of MVPA during PE sessions, focusing on demographic, psychological, and behavioral variables.

## 2 Methods and materials

### 2.1 Study design and setting

This cross-sectional study was conducted between October and December, 2024, in universities of Zhejiang Province, China.

### 2.2 Sampling and sample size

A total of 18 universities were randomly selected from the provincial list of accredited higher education institutions using proportional stratified sampling according to enrollment size. Within each selected university, cluster sampling at the class level was applied: classes comprising second-year undergraduates were randomly chosen, and all students from these years present in the selected classes were invited to participate. Second-year undergraduate students were selected as the study population for several reasons. At this stage, students have completed their first year of university, allowing them to be familiar with the academic environment and campus routines, yet they are still enrolled in mandatory PE courses, ensuring consistent exposure to structured activity. This makes them an ideal group for objectively assessing activity levels during PE lessons. Additionally, selecting second-year students minimizes self-selection bias that may occur in later years when PE participation becomes voluntary, allowing for a more representative assessment of typical student behavior. Moreover, second-year students are generally available and present in scheduled classes, facilitating accurate data collection and high response rates.

The minimum required sample size was estimated using G*Power (3.1.9.7) for regression (fixed model, R^2^ ≠ 0), with *α* = 0.05, power = 0.95, assumed small effect size f^2^ = 0.02. This calculation yielded 1,341 participants, which was increased to 1,610 to account for an anticipated 20% non-response/missing data rate.

Eligibility criteria included enrollment in the second year of undergraduate study. To reduce confounding, students in health-related majors (e.g., medicine, nursing, public health, PE) were excluded. Additional exclusion criteria were: severe mobility limitations (e.g., inability to participate in moderate physical activity for at least 10 min), and attendance at university fewer than 3 days per week.

### 2.3 Data collection instruments

Financial status was self-rated on a five-point Likert scale ranging from “very poor” (1) to “very good” (5). Due to low frequency in extreme categories, responses were collapsed into three categories: good (very good and good), acceptable (acceptable), and poor (very poor and poor) (12). Self-perceived financial condition has been widely used in youth health research as a valid proxy for actual socioeconomic status (13), especially where objective financial data is difficult to obtain.

BMI categories were recalculated based on the China CDC guidelines (underweight: <18.5; normal: 18.5–23.9; overweight: 24.0–27.9; obese: ≥28) (14). Height and weight were measured by using standardized procedures, and BMI was subsequently calculated. For regression analyses, BMI was dichotomized as “non-obese” (BMI < 28.0 kg/m^2^) and “obese” (BMI ≥ 28.0 kg/m^2^).

To objectively assess activity levels during PE lessons, ActiGraph wGT3X-BT accelerometers were used. Given that longer epoch lengths can underestimate vigorous activity and potentially overestimate MVPA by merging structured activity bouts, data were collected using 1-s epochs (15–17). Following each measurement session, the raw ActiGraph files were downloaded and processed using ActiLife software (version 6.5). Time spent at or above the moderate-intensity threshold (≥ 2,800 counts per minute) was classified as MVPA (18). The proportion of each PE lesson spent in MVPA was calculated by dividing the average MVPA time (in minutes) by the total lesson duration and expressing it as a percentage.

Physical activity was measured using the short version of the International Physical Activity Questionnaire (IPAQ-SF), which includes items on the frequency and duration of walking, MVPA, and screen time during the past 7 days. Each activity was assigned a metabolic equivalent (MET) value: 3.3 for walking, 4.0 for moderate-intensity, and 8.0 for vigorous-intensity activity. Total physical activity was calculated in MET-minutes/week. Participants were classified as physically inactive if they reported <600 MET-minutes/week, in line with previous studies (19, 20). Screen time was assessed via the IPAQ-SF item on time spent sitting during a typical weekday. The Chinese version of the IPAQ-SF has demonstrated acceptable test–retest reliability (r > 0.70) (21), and in the current study, it showed good internal consistency (Cronbach’s alpha = 0.82).

Sleep quality was assessed using the Chinese version of the Pittsburgh Sleep Quality Index (CPSQI), a 19-item self-report questionnaire (22). The 19 items are combined to form seven component scores, including subjective sleep quality, sleep latency, sleep duration, habitual sleep efficiency, sleep disturbance, sleep medication use, and daytime dysfunction (22). Each component score ranges from 0 to 3. Specifically, item 6 and item 4 contribute to the components subjective sleep quality and sleep duration, respectively; sleep latency is formed by items 2 and 5a; habitual sleep efficiency is formed by items 1, 3, and 4; sleep disturbance is formed by items 5b–j; sleep medication use is formed by item 7; and daytime dysfunction is formed by items 8 and 9 (22). The seven component scores are summed to yield a global PSQI score ranging from 0 to 21. In Chinese populations, a cutoff of 8 is commonly used to indicate poor sleep quality, with higher scores reflecting worse sleep (23). The Chinese version of PSQI has demonstrated good reliability among undergraduate students (23, 24). In the current study, Cronbach’s *α* for the scale was 0.702, and composite reliability for the total PSQI score was 0.79.

Mental health was assessed using the Chinese Mental Health Value Scale (CMHVS), a culturally-informed self-report instrument developed and validated to capture attitudes, beliefs, and values related to psychological well-being in Chinese populations (25). The CMHVS consists of 35 items organized into seven subscales: Expected Self, Relating to Others, Life Principles, Family, Purpose and Meaning, Achievement, and Communication. Each item is rated on a 5-point Likert scale ranging from 1 (strongly disagree) to 5 (strongly agree). Subscale scores are calculated by summing the relevant items, and a total score is computed by summing all 35 items. Total scores thus range from 35 to 175, with higher scores indicating stronger endorsement of health-promoting psychological values. The scale has demonstrated excellent internal consistency (Cronbach’s *α* = 0.96) and strong construct validity, supporting its use as a reliable measure of culturally-relevant mental health among Chinese university students (25).

Smoking status was evaluated using a four-category item: (1) never smoked regularly, (2) former smoker, (3) occasional smoker, and (4) daily smoker. This classification is supported by prior behavioral health surveys (26). For regression model, responses were further recoded into two categories: current smokers (occasional or daily) and non-smokers (never and former smokers) (26).

### 2.4 Data collection

Data were collected using structured self-administered questionnaires and objective accelerometer measurements. All participants provided written informed consent. Ethical approval was obtained from the Medical Research Ethics Committee of Zhejiang University (No. ZHU-20240209-51).

### 2.5 Statistical methods

Quantitative variables, including BMI, proportion of PE lesson spent in MVPA, total physical activity (MET-minutes/week), total CMHVS score, and PSQI global score, were summarized as means and standard deviations (M ± SD). Categorical variables, including gender, self-perceived financial condition, smoking status, and BMI category, were expressed as frequencies and percentages. To compare differences between female and male students, independent-sample t-tests were used for continuous variables, while Pearson’s chi-square tests were applied for categorical variables.

To address the primary research aim, regression analyses were conducted in two stages. First, multiple linear and logistic regression models were used to examine predictors of key health-related outcomes, including obesity (BMI ≥ 28, logistic regression), physical inactivity (total PA < 600 MET-min/week, logistic regression), poor sleep quality (PSQI >5, logistic regression), smoking status (current smoker, logistic regression), and mental health (CMHVS total score, linear regression). To minimize confounding, all models were adjusted for relevant covariates, including gender, BMI, financial condition, sleep quality, and mental health, depending on the outcome under study.

Second, to identify determinants of MVPA proportion in PE, a multiple linear regression model was conducted with MVPA proportion in PE lessons as the dependent variable. Potential predictors included BMI, gender, financial condition, sleep quality (PSQI score), and mental health (CMHVS score). A stepwise method was applied, with entry and removal criteria of *p* < 0.05 and *p* > 0.10, respectively. For linear regression models, assumptions of normality, homoscedasticity, and multicollinearity (tolerance >0.1, VIF < 10) were tested. For logistic regression models, the Hosmer–Lemeshow test was used to assess goodness-of-fit. The use of both objective accelerometer measures and validated self-report instruments further reduced the risk of measurement bias.

All analyses were conducted using SPSS version 26.0 (IBM Corp., Armonk, NY, United States), with a two-tailed *p*-value <0.05 considered statistically significant.

## 3 Results

**Figure 1:**
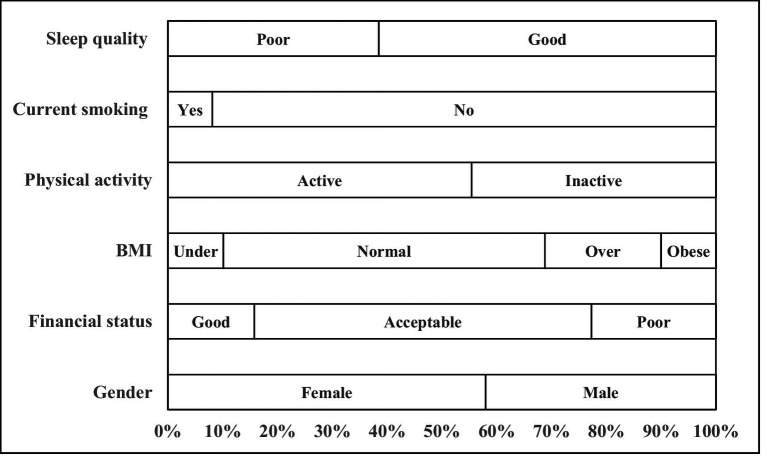
The characteristics of the participants.

A total of 2,117 valid questionnaires were collected from 2,953 distributed, yielding a response rate of 71.68% (age: 20.59 ± 0.62 years; female: 57.96%). Figure 1 shows the characteristics of the participants. 15.77% of students rated their financial status as good. 31.22% of students were overweight and obese (BMI ≥ 24) and 55.41% met the recommended physical activity level. The proportion of PE lesson time spent in MVPA was 48.62% ± 12.31%. Most students were non-smokers (91.87%) and 38.5% of students classified as having poor sleep quality. The mean daily screen time was 5.28 ± 1.12 h and mental health score was 137.47 ± 16.73, indicating generally positive mental health values in this population.

Gender-based comparisons revealed significant differences across several variables (Table 1). Male students reported higher mental health scores and had a higher mean BMI than females (*p* < 0.001). They were also more physically active than females, both in meeting activity guidelines and in the proportion of PE class time spent in MVPA (*p* < 0.001). Also, smoking was far more common among males and they reported longer daily screen exposure (*p* < 0.001). Females, however, exhibited significantly poorer sleep quality (*p* < 0.001).

**Table 1:** Gender differences in study variables.

Variable	Male (*n* = 890)	Female (*n* = 1,227)	Test statistic	*p*
BMI (kg/m^2^) (Mean ± SD)	23.78 ± 3.47	21.63 ± 3.25	12.53	< 0.001
Physically active (%, n)	62.92%, *n* = 560	49.96%, n = 613	35.22	< 0.001
MVPA proportion during PE (Mean ± SD)	52.1 ± 12.7	46.1 ± 11.8	10.02	< 0.001
Current smoking (%, n)	14.6%, n = 130	3.42%, n = 42	102.71	< 0.001
Daily screen time (hours), (Mean ± SD)	5.56 ± 1.18	5.06 ± 1.06	9.18	< 0.001
Sleep quality (Mean ± SD)	7.32 ± 3.08	8.12 ± 3.2	−6.47	< 0.001
Mental health (Mean ± SD)	138.9 ± 16.4	136.4 ± 16.9	5.82	< 0.001

Multiple linear regression was used to examine predictors of mental health (CMHVS total score; Table 2). The overall model was statistically significant, *F*(5, 1,167) = 25.65, *p* < 0.001, explaining 9.9% of the variance in mental health (R^2^ = 0.099). Among the predictors, higher total physical activity (*β* = 0.17, *p* = 0.003) and greater MVPA during PE lessons (*β* = 0.19, *p* = 0.003) were associated with better mental health, while longer screen time (*β* = −0.14, *p* = 0.009) was associated with poorer mental health.

**Table 2:** Multiple linear regression analysis predicting mental health scores from physical activity, screen time, MVPA during PE, gender, and financial status.

Predictors	Dependent variable: mental health			
	*Β*	SE	T	*p*
Gender	0.03	0.04	1.01	0.309
Financial status	−0.01	0.03	−0.63	0.527
Total physical activity	0.17	0.06	3.14	0.003*
Screen time	−0.14	0.06	−2.57	0.009*
MVPA proportion in PE lessons	0.19	0.07	3.02	0.003*

Model summary: R^2^ = 0.099, Adjusted R^2^ = 0.093, F(5, 1,167) = 25.65, *p* < 0.001.

The univariate and multivariate logistic regression analyses were conducted to examine predictors of obesity (BMI ≥ 28), physical inactivity (total physical activity <600 MET-min/week), poor sleep quality (PSQI ≥8), and current smoking status (Table 3). In the multivariate models, a higher proportion of MVPA during PE lessons was significantly associated with lower odds of obesity (adjusted OR = 0.41, 95% CI: 0.31–0.59, *p* < 0.001), physical inactivity (adjusted OR = 0.38, 95% CI: 0.27–0.52, *p* < 0.001), and poor sleep quality (adjusted OR = 0.72, 95% CI: 0.57–0.91, *p* = 0.004). Also, male students had higher odds of obesity (OR = 3.14, 95% CI: 2.22–4.43, *p* < 0.001) and smoking (OR = 2.02, 95% CI: 1.36–3.01, *p* = 0.001).

**Table 3:** Logistic and linear regression analyses predicting health-related outcomes.

Outcome	Predictor	Unadjusted OR (95% CI)	Adjusted OR (95% CI)	*p*-value
Obesity	MVPA proportion in PE	0.46 (0.34–0.62)	0.41 (0.31–0.59)	<0.001
	Male (vs Female)	3.27 (2.31–4.62)	3.14 (2.22–4.43)	<0.001
	Financial status (acceptable and poor vs. good)	0.95 (0.71–1.28)	0.97 (0.72–1.31)	0.85
		0.97 (0.73–1.25)	0.95 (0.70–1.21)	0.119
Physical inactivity	MVPA proportion in PE	0.40 (0.29–0.55)	0.38 (0.27–0.52)	<0.001
	Male (vs Female)	0.91 (0.73–1.15)	0.88 (0.70–1.11)	0.30
	Financial status (acceptable and poor vs. good)	1.08 (0.82–1.42)	1.06 (0.80–1.40)	0.68
		1.1 (0.85–1.41)	1.07 (0.81–1.4)	0.23
Poor sleep	MVPA proportion in PE	0.75 (0.60–0.93)	0.72 (0.57–0.91)	0.004
	Male (vs Female)	1.12 (0.88–1.43)	1.09 (0.85–1.40)	0.48
	Financial status (acceptable and poor vs. good)	0.97 (0.73–1.29)	0.96 (0.72–1.28)	0.78
		0.95 (0.72–1.28)	0.93 (0.7–1.24)	0.322
Smoking (current)	MVPA proportion in PE	0.92 (0.59–1.43)	0.94 (0.60–1.46)	0.78
	Male (vs Female)	2.08 (1.40–3.08)	2.02 (1.36–3.01)	0.001
	Financial status (acceptable and poor vs. good)	0.88 (0.53–1.46)	0.90 (0.54–1.49)	0.67
		0.91 (0.57–1.48)	0.89 (0.53–1.46)	0.209

Also, to identify determinants of MVPA proportion in PE lessons, a multiple linear regression analysis was performed with MVPA proportion as the dependent variable (Table 4). The overall model was significant (*F*(5, 2,330) = 18.62, *p* < 0.001), explaining 9.5% of the variance in MVPA proportion (R^2^ = 0.095). Among the predictors, higher BMI (*β* = −0.11, *p* < 0.001), female gender (*β* = −0.14, *p* < 0.001), poorer sleep quality (PSQI score, *β* = −0.09, *p* = 0.003), and lower mental health scores (*β* = 0.11, *p* = 0.002) were associated with a significantly lower proportion of MVPA during PE. Financial condition was not a significant predictor (*p* = 0.163).

**Table 4:** Multiple linear regression analysis predicting MVPA proportion in PE lessons from BMI, sleep quality, mental health, gender, and financial status.

Predictors	Dependent variable: MVPA proportion in PE lessons			
	Β	SE	T	*p*
BMI	−0.11	0.03	−6.14	<0.001
Gender (female)	−0.14	0.03	−7.32	<0.001
Financial condition	0.02	0.02	1.38	0.163
Sleep quality (PSQI)	−0.09	0.03	−2.87	0.003
Mental health (CMHVS)	0.11	0.03	3.09	0.002

Model summary: R^2^ = 0.095, Adjusted R^2^ = 0.092, F(5, 2,330) = 18.62, *p* < 0.001.

## 4 Discussion

University PE programs have long been recognized as a strategic platform for promoting the physical and mental health of students. However, evidence regarding their effectiveness in shaping sustainable health-related behaviors among university populations, especially in China, remains. This study aimed to address this gap by examining the association between participation in PE classes and mental health and some health-related behaviors, including physical inactivity, obesity, and smoking. These findings may indicate the importance of evaluating PE classes not only in terms of physical outcomes, but also as a means of promoting students’ overall health.

This study found that higher total physical activity and a greater proportion of MVPA during PE lessons were associated with better mental health, whereas longer screen time showed a negative association. Gender and self-perceived financial status did not appear to influence mental health in this sample. Therefore, implementing strategies to increase MVPA in PE classes and reduce screen time may help improve students’ mental health.

Although causality cannot be inferred from this cross-sectional study, several plausible mechanisms may explain these relationships. Regular physical activity can enhance mood and emotional regulation through neurobiological pathways, including increased release of endorphins and neurotransmitters such as serotonin and dopamine (27, 28). Active participation during PE lessons may further promote self-efficacy, social engagement, and a sense of accomplishment, which can contribute to psychological well-being (29, 30). Conversely, prolonged screen time may replace opportunities for physical activity, limit social interactions, and induce mental fatigue or stress, potentially detracting from mental health (31, 32). The mechanisms suggested suggest that PE classes may be able to be purposefully designed to help foster self-efficacy and social interactions, while broader academic programs could also play a role in more effectively managing students’ screen use.

Our findings show that a higher proportion of MVPA during PE lessons was associated with lower odds of obesity, physical inactivity, and poor sleep quality. Specifically, students engaging in more MVPA appeared less likely to be obese, less likely to report physical inactivity, and less likely to experience poor sleep. While causality cannot be inferred from this observational study, several mechanisms may help explain these patterns. Higher MVPA during PE could reflect greater overall activity levels, which may contribute to energy balance and body weight regulation (33), consistent with prior research linking active university-based physical activity to lower obesity prevalence (34, 35). Likewise, students who are more active during PE might be more inclined to maintain other forms of daily activity, thereby reducing sedentary behavior and inactivity. These results may indicate that PE programs can be used as preventive strategies against obesity and inactivity while also helping to improve sleep hygiene among students.

The observed association between higher MVPA and better sleep quality could be mediated through multiple physiological and behavioral pathways. Physical activity may enhance sleep homeostasis by increasing sleep drive, improving circadian rhythm regulation, and reducing stress or anxiety, all of which can positively influence sleep efficiency (36, 37). These pathways are supported by previous studies demonstrating links between regular physical activity and improved sleep metrics in university students (38–40). Encouraging more intense activities in PE classes may be a practical approach to improving students’ sleep quality and academic performance.

No significant association was found between MVPA proportion and smoking status, suggesting that the behavioral patterns influencing substance use may be distinct from those shaping physical activity (41). Social and environmental influences, peer behaviors, and personality traits may play stronger roles in smoking behaviors than participation in PE (42). This may indicate that smoking status in universities requires targeted interventions in the areas of public health and social issues that go beyond the framework of PE programs.

Gender differences were evident for obesity and smoking, with male students showing higher odds of both outcomes. This aligns with prior literature reporting sex-based differences in body composition (43, 44), activity preferences, and risk-taking tendencies during university (45–47). However, gender was not significantly associated with physical inactivity or poor sleep, indicating that the potential benefits of MVPA during PE lessons may be experienced similarly by both sexes in these domains. Gender-specific interventions, particularly in the areas of obesity and smoking among male students, may be necessary, while the benefits of PE classes could be promoted equally among both sexes.

Self-perceived financial status did not significantly predict any of the examined outcomes, suggesting that the relationships between MVPA and health-related behaviors operate independently of students’ perceived economic resources. Overall, these results highlight that higher engagement in MVPA during PE is consistently associated with favorable patterns in weight status, activity levels, and sleep, while emphasizing the need for cautious interpretation due to the cross-sectional design of the study.

One of the findings of the present study was that higher BMI, female gender, poorer sleep quality, and lower mental health were associated with a lower proportion of MVPA during PE lessons among university students. These associations suggest that multiple interrelated factors may influence students’ engagement in moderate-to-vigorous activity. Higher BMI may reduce participation through both physiological and psychological pathways: excess body weight can increase fatigue and perceived exertion during activity (48), while also lowering perceived physical competence and self-efficacy (49). Female students may engage in lower MVPA due to sociocultural influences, gender norms, and differences in motivation or enjoyment during PE classes, as observed in previous studies among young adults (49). Poor sleep quality can impair alertness, energy metabolism, and physical performance, potentially reducing activity intensity, while suboptimal mental health may affect motivation, emotional regulation, and the ability to cope with physical challenge (50, 51). These findings may indicate that interventions should not only focus on promoting MVPA in general, but also need to provide more targeted supports for students with higher BMI, girls, and those experiencing sleep or mental health problems.

These findings align with prior research indicating that physical, psychological, and social factors jointly shape activity behaviors in higher education settings. Understanding these determinants can inform targeted strategies, such as tailored PE programs, supportive interventions for students with higher BMI, initiatives to improve sleep hygiene, and mental health support programs, all aimed at enhancing engagement in MVPA among university students.

Several limitations of the present study should be acknowledged. First, the cross-sectional design precludes causal inferences; although significant associations were observed, the directionality of relationships cannot be established. Second, some measures relied on self-report, which may introduce reporting bias, particularly for physical activity, screen time, and mental health. Objective measurements could enhance accuracy in future studies. Third, the sample consisted solely of university students from a single region, limiting the generalizability of findings to other populations or cultural contexts. Fourth, while multiple relevant predictors were included, unmeasured factors such as dietary habits, academic stress, or social support could also influence both MVPA participation and mental health. Finally, the proportion of variance explained in the models was modest, suggesting that other determinants remain unaccounted for. Although we adjusted for several potential confounders, such as gender, BMI, sleep quality, and financial status, residual confounding from unmeasured variables (e.g., dietary habits, academic stress, or social support) cannot be ruled out. Future research should employ longitudinal and interventional designs, incorporate more objective measures, and explore additional contextual and behavioral variables to build stronger evidence.

## 5 Conclusion

In conclusion, the present study shows that both overall physical activity and participation in MVPA in PE classes are positively associated with better mental health among university students. In contrast, higher BMI, female gender, poor sleep quality, and poor mental health are associated with reduced participation in MVPA in PE classes. These findings emphasize the multifactorial nature of physical activity-related behaviors in higher education settings and suggest that interventions aimed at improving mental health and physical activity should simultaneously address physical, psychological, and behavioral factors. Targeted strategies—such as promoting inclusive PE environments, supporting students with higher BMIs, improving sleep quality, and providing mental health resources—may help optimize physical activity participation and health promotion in the university population.

At the same time, this study suggests avenues for future research. Longitudinal, intervention-based designs are essential to determine causal relationships, and the use of more objective instruments to measure health behaviors can reduce the limitations of self-reported data. Also, larger studies across multiple educational centers can help increase the generalizability of the results, and examining additional factors such as dietary habits, academic stress, and social support also merits further consideration.

From an educational perspective, the results of this study emphasize the potential of PE classes as a platform for comprehensive health promotion on campus. Designing PE curricula that maximize opportunities for MVPA, enhance enjoyment, and address barriers for students with high BMI or mental health challenges may lead to greater participation. Incorporating educational elements such as sleep hygiene and stress management into PE programs can also improve outcomes. By aligning PE classes with campus-wide health initiatives, we can create supportive environments that promote both physical activity and mental health.

## Data Availability

The raw data supporting the conclusions of this article will be made available by the authors, without undue reservation.
